# Removal Ability and Resistance to Cinnamic and Vanillic Acids by Fungi

**DOI:** 10.3390/microorganisms8060930

**Published:** 2020-06-19

**Authors:** Barbara Speranza, Francesca Cibelli, Antonietta Baiano, Antonia Carlucci, Maria Luisa Raimondo, Daniela Campaniello, Ilaria Viggiani, Antonio Bevilacqua, Maria Rosaria Corbo

**Affiliations:** Department of the Science of Agriculture, Food and Environment, University of Foggia, 71122 Foggia, Italy; barbara.speranza@unifg.it (B.S.); francesca.cibelli@unifg.it (F.C.); antonietta.baiano@unifg.it (A.B.); antonia.carlucci@unifg.it (A.C.); marialuisa.raimondo@unifg.it (M.L.R.); daniela.campaniello@unifg.it (D.C.); ilaria2710@alice.it (I.V.)

**Keywords:** aromatic acids, fungi, antifungal effect, removal, growth kinetic

## Abstract

Twelve fungal strains were assayed to investigate their resistance to cinnamic and vanillic acids and their ability to remove these compounds from a liquid medium. In a first step, the effect of the two aromatic acids (1 g/L) on the fungal growth kinetic was studied. The results were modelled through a logistic like function (Dantigny equation) to estimate *τ*, which is the time to the half-maximum colony diameter. The key findings of this part were as follows: (i) generally, cinnamic acid exerted a stronger effect than vanillic acid; (ii) aromatic acids exerted a delay on the growth of some fungi and only one strain (*Athelia rolfsii*) was completely inhibited. In the second part, fungi were assayed to investigate their ability to remove cinnamic and vanillic acids (ca. 350 mg/kg) from liquid media at pH 3.5. The results indicated that the most efficient fungi were *Aspergillus niger* and *Lasiodiplodia theobromae*.

## 1. Introduction

In Apulia (Southern Italy), Olive Mill Wastewaters (OMWW) are the most important agro-food effluents and are known as a good source of some bioactive compounds [1]. The habit of releasing them in agricultural soil, which is largely widespread among producers, could significantly modify the microbiological, chemical, and physical profiles of soil and the rhizosphere due to their content of phenolics and other aromatic compounds.

According to Asses et al. [2] the average composition of OMWW is 83–96% water, 3.5–15% organic matter, and 0.5–2% mineral salts. 

Mantzavinos and Kalogerakis [3] reported that the acids commonly found in OMWW can be divided into three major families: cinnamic acid derivatives, benzoic acid derivatives, and tyrosol derivatives. Cinnamic (coumaric, caffeic, and ferulic acids) and benzoic acids (hydroxybenzoic, vanillic, gallic, and protocatechuic acids) derivatives are amongst those compounds commonly present in OMWW at concentrations that may vary from as low as 0.05–0.2 g/L to as high as 10 g/L depending on the type and origin of the effluent [4].

Phenols, acids, and other aromatic compounds can be removed by some fungi, thanks to some enzymes [5] which are able to degrade cyclic ring compounds.

In a preliminary study [6,7], some fungal strains were studied for their ability to grow on minimal media supplemented with different amounts and kinds of OMWW, but OMWW can contain simple acids (low molecular weight compounds) or complex phenols (high molecular weight compounds produced from the degradation of oleuropein). The results pointed out the ability of some strains to grow in minimal media containing 5–15% OMWW; however, for an effective design of a bioremediation tool it is important to know the effect of each compound of OMWW or of some representative molecules on fungal growth. 

Therefore, the main goal of this research was to evaluate the fungal growth and removal ability on two different classes of compounds (hydroxybenzoic acids and several phenols) by using two representative compounds. Vanillic acid was used as a representative of the class of hydroxybenzoic acids. Cinnamic acid is a monocarboxylic acid that plays a central role in the biosynthetic pathway leading to phenyl-propanoids, coumarins, lignans, isoflavonoids, flavonoids, stilbenes, anthocyanins, and tannins. It is a precursor of several phenolic acids, being involved in the metabolic pathways of compounds such as *p*-coumaric, caffeic, and chlorogenic acids, and was used as a representative of the class of cinnamic acids.

This research was divided in two different steps: (i) the study of fungal resistance and (ii) the evaluation of the removal ability towards vanillic and cinnamic acids in a simple liquid medium.

## 2. Materials and Methods 

### 2.1. Fungi

Twelve reference fungal species from a fungal pool assayed on different OMWWs in previous researches [6,7] (Table 1) were studied to assess their growth on media containing either vanillic (code 94770, purum ≥ 97.0%, HPLC, Sigma-Aldrich) or cinnamic (code 133760, 97%, Sigma-Aldrich) acids.

### 2.2. Growth on Minimal Media Containing Cinnamic or Vanillic Acid

The acids (1 g/L) were added to a minimal medium (15 g/L Agar Technical n.3; Oxoid Ltd., Basingstoke, UK). Then, the prepared media were sterilized at 121 °C for 20 min and then poured into sterile 55 mm-diameter Petri dishes. The fungi were aseptically inoculated using 2 mm mycelium plugs cut from the colony margins of 7–10-day-old cultures. The absence of other carbon sources allowed us to test the ability of fungi to use cinnamic and vanillic acids as their sole carbon sources. In fact, previous works have highlighted that most fungi are able to partially or totally degrade these aromatic acids [8,9,10].

The media inoculated were then incubated at 23 ± 2 °C in the dark. The same fungal cultures were grown on Agar (adjusted to pH 3.5) without acids as controls. The diameters of the fungal colonies were measured at intervals of 48 h over 21 days in comparison with those grown on Agar control plates. The experiment was repeated twice, and each time 3 petri dishes were inoculated for each fungal strain. The fungal growth was modeled using a logistic equation as modified by Dantigny et al. [11] and given in Equation (1):

(1) $$D=\frac{D_{\max}}{1+\exp[k(\tau -t)]},$$

where *D* is the diameter of the fungal colony at the chosen time, *D*_max_ is the maximum diameter of the fungal colony (here set as 55 mm; i.e., the diameter of the plates), *k* is the rate of fungal growth (mm/day), τ is the time to attain half *D*_max_ (days), and *t* is the chosen time of the analysis (days).

In order to assess the fitting parameters for all the different fungal species used, the dataset fitting was performed using the Statistica for Windows software (version 12.0; Statsoft, Tulsa, OK, USA) with a least-squares approach. The significant differences in the parameter τ were investigated through a one-way ANOVA and Tukey’s test (*p* < 0.05).

Then, the τ values were standardized as Δτ, given in Equation (2):

Δτ = τ − τ_C_, (2)

where τ and τ_C_ are the τ values from the media supplemented with acids and from the control medium (agar without acids), respectively.

A positive Δτ indicates the inhibition of fungal growth due to the presence of the two acids, whereas a negative Δτ indicates that the fungal growth was promoted.

The Δτ values were then used as the dependent variable for a multi-factorial analysis of variance (MANOVA) according to two different factors as categorical predictors (i.e., independent variables): the fungus (F), and the acid used (Phe).

### 2.3. Evaluation of Fungal Biomass on Cinnamic and Vanillic Acids

Twelve flasks containing 500 mL of distilled water were added with about 350 mg/Kg of vanillic or cinnamic acids. The solutions obtained were subjected to sonication (ULTRASONIC FALC 4300 MH) for 10 min in order to obtain a complete acid dissolution (power 20%; the total net power was 130 W). The pH of the solutions ranged from 3.34 to 3.65. Aliquots of 49 mL were transferred into 100 mL flasks and sterilized at 121 °C for 20 min. After cooling in the dark, the samples were inoculated with 1 mL of a conidial suspension (10^3^ CFU/mL, colony forming units) of each fungal species. The flasks were then incubated at 25 °C for 21 days in the dark.

After 21 days, with the aid of a vacuum pump all the liquid cultures were filtered through sterile Buckner funnels (70 mm diameter) in order to retain the mycelium on Miracloth paper (Calbiochem, CA) previously conditioned in a stove at 105 °C for 24 h. The weight of each fungal mycelium was determined after drying in the stove at 105 °C.

For each liquid fungal culture, the color (Tristimulus Colorimeter, Konica Minolta, Japan) and pH were evaluated. The experiments were performed at least over two independent batches. The results were analyzed as the amount of biomass produced and submitted to ANOVA and MANOVA.

### 2.4. Analysis of Vanillic and Cinnamic Acids

An HPLC analysis of the cinnamic and vanillic acid concentrations in the liquid medium samples from the experiments described in Section 2.3 was carried out according to Gambacorta et al. [12] using a HPLC binary system (Agilent, model G1311A, Santa Clara, CA, USA) equipped with a 7725 Rheodyne injector, a 20 μL sample loop, adiode array detector (Agilent, model G1315Bm), and a Chem Station integrator (Agilent) for data acquisition. The stationary phase was a Nova-Pack C18 analytical column (150 mm length, 3.9 mm i.d.) with a particle size of 4 μm (Waters, Milford, MA, USA). The mobile phases for chromatographic analysis were (A) water:acetic acid (98:2, *v*/*v*) and (B) methanol:acetonitrile (1:1, *v*/*v*) at a constant flow rate of 1 mL/min. The gradient program of solvent was as follows: 0 to 30 min 100% A; 30 to 45 min 70% A; 45 to 55 min 50% A; 55 to 65 min 40% A; 65 to 75 min 0% A. The compound identification was carried out by comparing the peak retention times with those obtained by the injection of pure standards. The concentrations were expressed as mg/L.

The experiments were performed at least over two independent batches. The results were analyzed as a decrease in phenols after 21 days and submitted to ANOVA and MANOVA.

## 3. Results and Discussion

### 3.1. Growth Kinetic on Solid Media Containing Cinnamic or Vanillic Acid

Fungal resistance was evaluated as “radial growth” on a solid medium containing either vanillic or cinnamic acid (1 g/L). The results are reported in Table 2.

*Lasiodiplodia theobromae* (**Lt**), *Phaeoacremonium parasiticum* (**Pp**), and *Diaporthe amygdali* (**Dg**), which are wood disease agents of several fruit trees [13,14,15,16], were not affected by any of the two acids, except for **Dg**, which showed an increase in τ in the presence of cinnamic acid (from 4.10 to 6.60 days).

*Colletotrichum gloeosporioides* (**Cg**) and *Alternaria alternata* (**Aa**), which are causal agents of anthracnose and black spot disease on olive drupes, respectively [17,18], were significantly affected by cinnamic acid, as evidenced by the significant increase in τ (from 3.85 to 5.01 days for **Cg** and from 3.05 to 5.88 days for (**Aa**). *Fusarium oxysporum* (**Fx**), a soil-borne fungus [19], and *Penicillium italicum* (**Pi**), a necrotrophic fungus [20], were not affected by the acids. *Aspergillus ochraceus* (**Ao**) and *Aspergillus niger* (**An**), which are soil borne fungi [3,21], were significantly affected by cinnamic acid, as highlighted by the significant increase in τ. Another soil-borne fungus, *Trichoderma* sp. (**Tch**), was significantly affected by both acids, with the τ decreased in the presence of vanillic acid and increased in the presence of cinnamic acid.

*Athelia rolfsii* (**Ar**) and *Rosellinia necatrix* (**Rx**) are known as two severe soilborne fungal pathogens of horticultural crops [22] and fruit trees [23]. The first was affected by both acids although in a different way, since it experienced complete inhibition in the presence of cinnamic acid, while τ increased from 3.00 to 6.31 days in the presence of vanillic acid. Instead, **Rx** was affected only by cinnamic acid, as indicated by the increase in τ from 4.17 to 7.89 days.

**Figure 1:**
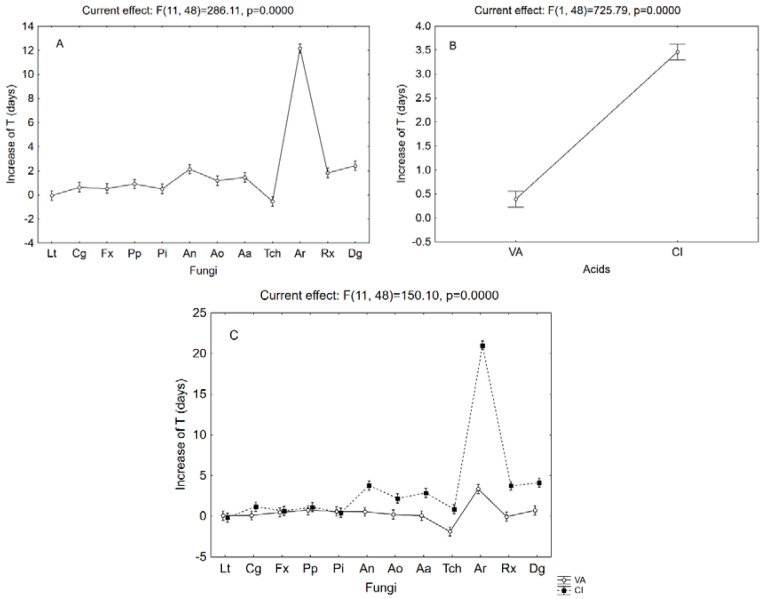
Effective hypothesis decomposition of the multi-factorial analysis of variance (MANOVA) on Δτ (increase in time to attain half maximum diameter) (days). Effects of fungi species (for abbreviations, see Table 1) on (**A**) acids, (**B**) interaction fungus* acid, (**C**) (VA, vanillic; CI, cinnamic acid). Vertical bars indicate 95% confidence intervals. The F-test and relative degrees of freedom are given at the top of each panel.

The analysis of τ pointed out a possible effect of cinnamic acid on fungal growth; however, this approach cannot be used to compare the kinetics of different fungi, because each fungus has a different trend, as inferred by the τ value of controls. Therefore, the results were preliminary standardized and used as Δτ (increase in the parameter compared to the control) to run a MANOVA and to weight the statistical effect of the fungi and acids. The Δτ was mainly affected by the acids used, followed by the fungal species and finally by the interactive term fungi vs. acids (data not shown). A second output of MANOVA is the decomposition of the statistical hypothesis, showing the quantitative effect of each predictor. Figure 1A shows the effect of the fungal species Generally, the effect of acids was light, as the mean increase in τ was 2 days or lower; only for **Ar** a higher mean effect was found (12 days).

As expected from the actual values, cinnamic acid exerted a stronger effect than vanillic acid (Figure 1B); however, Figure 1C shows the actual trend and an effective comparison of the bioactivity of acids. The mean Δτ experienced a three-class trend for cinnamic acid: group 1: Δτ, 0 days (**Lt**, **Cg**, **Fx**, **Pp**, **Pi**, **Tch**); group 2: Δτ < 4 days (**An**, **Ao**, **Aa**, **Rx**, **Dg**); group 3: inhibition, at least for 21 days (**Ar**).

Generally, vanillic acid did not affect fungal growth, while a delay was found with cinnamic acid for most strains and complete inhibition was found for a single strain for at least 21 days (**Ar**). Some studies on the antioxidant and antimicrobial properties of cinnamic acid and its derivatives just focused on the potential of these compounds in pharmacological studies aimed at discovering novel antifungal drugs. Due to their low cytotoxicity, the inhibitory and antifungal properties of cinnamic acid derivatives have been further explored in the food and cosmetics industries [24,25].

Cinnamic acid, chlorogenic acid, p-coumaric acid, and some other cinnamic acid derivatives were active against several fungal pathogens [26,27,28,29]. An analogue of cinnamic acid, (E)-3-(4-methoxy-3(3-methylbut-2-enyl)phenyl) acrylic acid, strongly inhibited the growth of *A. niger*, *A. terreus*, and *A. flavus*, with a similar effect as miconazole [30]. The effect relies upon the competitive inhibition of benzoate 4-hydroxylase CYP53A15 [31] or the disintegration of the plasma membrane and the production/accumulation of ROS (reactive oxygen species) inside cells [32].

The difference in the bioactivity of vanillic and cinnamic acids as well as the different response of fungal species and genera could be linked to differences in the solubility and composition of the plasma membrane [33]; in addition, the lower bioactivity of vanillic acid could also rely on the production by many fungi of the enzyme vanillate hydroxylase, which decarboxylates vanillic acid and produces hydroquinones [34]. In addition, the metabolic pathway for the degradation of cinnamic acid suggested by Lubbers et al. [9] points out the existence of a complex multi-step process and the presence, in an intermediate step, of benzoic acid, which is a well-known antifungal compound.

### 3.2. Removal of Cinnamic or Vanillic Acid in Liquid Media

All the strains were resistant to aromatic acids; therefore, they were all used in the second step. The numerical results are reported in Table 3.

The results of Table 3, expressed as reduction of acids in the media, were used as for a MANOVA to point out the individual effect of each factor and the possible interactive terms. The most significant term was the kind of acid, followed by the fungal strain and the interaction fungal strain vs. acid. As for the experiments on the antimicrobial activity of cinnamic and vanillic acids, the decomposition of the statistical hypothesis highlighted the effective trends for each predictor.

**Figure 2:**
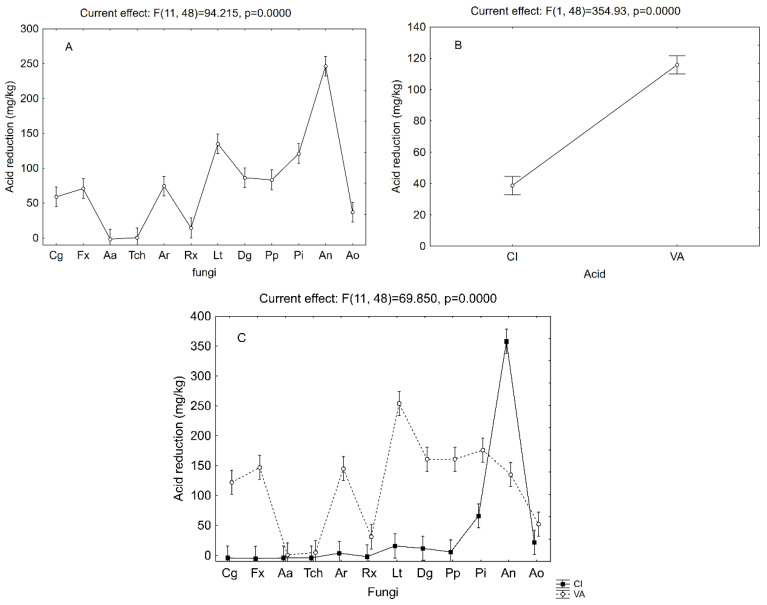
Effective hypothesis decomposition of MANOVA on the decrease in the acids (mg/kg). Effects of fungi species (for abbreviations, see Table 1) on (**A**) acid vs. pH (**B**) and fungi vs. acid (**C**) (CI, cinnamic; VA, vanillic acid). Vertical bars indicate 95% confidence intervals. The F-test and relative degrees of freedom are given at the top of each panel. Contr., liquid medium without fungi.

Figure 2A shows the effect of fungal strain independently on the kind of acid; the most efficient strain was **An** (mean efficiency 330 mg/kg); followed by **Lt** and **Pi** (mean efficiency 130–140 mg/kg); and **Cg**, **Fx**, **Ar**, **Dg,** and **Pp** (50–100 mg/kg). The decomposition for the effect of the acid is shown in Figure 2B; fungi showed a preference toward vanillic acid with a mean efficiency of 110 mg/kg.

The behavior of *A. niger* confirms many literature reports regarding the ability of *Aspergillus* spp. to act as an efficient tool for bioremediation [6,35,36,37]. *Lasiodiplodia theobromae* is well known for its phyto-pathological effects [38]; it was also proposed for the extraction of lipases to be used for the production of biodiesel from coconut oil [39]. However, to the best of our knowledge this is the first report on the ability of this fungus to remove aromatic acids from liquid media.

The decomposition of the statistical hypothesis (Figure 2C) for the interaction of fungi vs. acids highlighted the preference of fungi for cinnamic or vanillic acids—i.e., the different removal abilities towards the two compounds. *Aspergillus niger* removed high amounts of cinnamic acid (340 mg/kg), followed by **Pi** (66 mg/kg). The other fungi acted only on vanillic acid or did not exert any effect. The highest reduction of vanillic acid was found for **Lt** (254 mg/kg); **Dg**, **Pp,** and **Pi** (161–176 mg/kg); **Cg**, **Fx**, **Ar**, and **An** (122–147 mg/kg); and **Ao** and **Rx**, which showed the lowest reduction (54 and 31 mg/kg, respectively). **Aa** and **Tch** did not exert significant effects.

**Figure 3:**
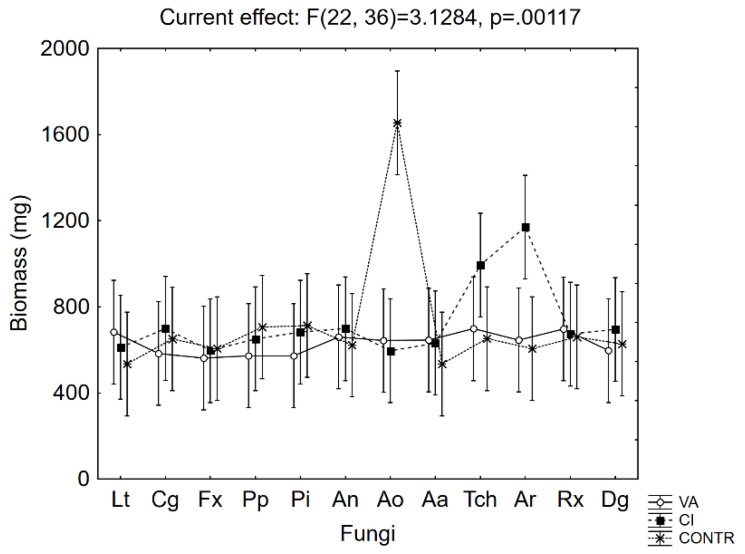
Effective hypothesis decomposition of MANOVA on biomass (mg). Interaction fungi vs. acids (for abbreviations, see Table 1). Vertical bars indicate 95% confidence intervals. The F-test and relative degrees of freedom are given at the top. Contr., liquid medium without acids.

Along with acid reduction, the biomass production was assessed (Figure 3); after 21 days, the effect of the acid was not significant (*p* > 0.05) for most fungi.

It is a matter of debate whether the reduction of acids was the result of adsorption or metabolization.

The metabolism of the aromatic acids object of this study depends on the species and perhaps on the strain origin. According to Guiraud et al. [8], the possible metabolic pathways involving vanillic acids are the following: (i) the demethylation of vanillic acid to protocatechuic acid by the vanillate demethylases (protocatechuic); (ii) the oxidative decarboxylation of vanillic acid to methoxy-hydroquinone by vanillate (decarboxylating) hydroxylase and further to hydroxyquinol (methoxy-hydroquinone pathway); (iii) the reduction of vanillic acid to vanillin and vanillic alcohol. The pathways (i) and (ii) occur in *Aspergillus ochraceus* and *Penicillium italicum*, which are known to produce protocatechuic acid and methoxy-hydroquinone. The pathway (ii) also occurs in *Aspergillus niger,* while *Trichoderma* spp. produces vanillin and vanillic alcohol through the (iii) pathway.

Concerning cinnamic acid, one of the most studied pathways is the non-oxidative decarboxylation of cinnamic acid to styrene. It occurs in fungi, such as *Aspergillus*, *Penicillium*, and *Trichoderma* [40,41,42]. When assayed OMWW, some fungal strains showed different trends, as the most resistant fungi were **Ao** and **Pp** [6]; however, **An** and **Lt**, which were the most promising strains in this research, also showed the ability to grow on media containing OMWW with a high content of phenol. This trend was attributed to the isolation source of the two strains (phenol-rich niches, that is, OMWWs and grapevine plants) probably responsible for an adaptive evolution towards resistance to phenols [6].

The removal of compounds such as cinnamic and vanillic acids is a complex process and could have strong practical implications in the bioremediation of agro-food effluents; this evidence was found for **An** and **Lt**, which were able to grow on OMWW and removed the highest amounts of aromatic acids. However, the results for **Ao** (low efficiency in removing aromatic acids, but able to grow on OMWW) also suggest that other factors and variables are involved.

The approach proposed in this paper (resistance and investigation on removal ability), as well as the use of a primary model (Dantigny) coupled with MANOVA could represent a useful method for the preliminary selection of promising fungal strains able to remove a compound from a liquid medium. Further investigations are required to elucidate the exact mechanism for each species/strain (adsorption and/or metabolization) in order to design an effective removal process.

## Figures and Tables

**Table 1:** Details of the fungal species used in this study. MC, molecular characterization; MOR, morphological and microscopic characterization; Dept. SAFE, Department of the Science of Agriculture, Food, and Environment, University of Foggia, Foggia, Italy.

Fungal Species	Host	Locality	Identification Tools	Reference or Collection	Abbreviation
*Lasiodiplodia theobromae*	*Vitis vinifera*	Cerignola, Foggia, Italy	MC	[13]	Lt
*Colletotrichum gloeosporioides*	*Olea europea*	Matino, Lecce, Italy	MOR	Dept.SAFE	Cg
*Fusarium oxysporum*	*Olea europea*	Presicce, Lecce, Italy	MC	Dept.SAFE	Fx
*Phaeoacremonium parasiticum*	*Olea europea*	Cerignola, Foggia, Italy	MC	[14]	Pp
*Penicillium italicum*	Soil	Stornarella, Italy	MOR	Dept.SAFE	Pi
*Aspergillus niger*	Oil-mill wastewater	Cerignola, Foggia, Italy	MOR	Dept.SAFE	An
*Aspergillus ochraceus*	Soil	Stornarella, Foggia, Italy	MOR	Dept.SAFE	Ao
*Alternaria alternata*	*Olea europea*	Cerignola, Foggia, Italy	MOR	[14]	Aa
*Trichoderma* sp.	Soil	Stornarella, Foggia, Italy	MOR	Dept.SAFE	Tch
*Athelia rolfsii*	*Solanum lycopersicum*	Stornarella, Foggia, Italy	MOR	Dept.SAFE	Ar
*Rosellinia necatrix*	*Olea europea*	Cerignola, Foggia, Italy	MOR	Dept.SAFE	Rx
*Diaporthe amygdali*	*Prunus dulcis*	Matino, Lecce, Italy	MC	Dept.SAFE	Dg

**Table 2:** Time to attain to half maximum diameter of the fungal colony *(D*_max_) (days) (mean ± standard error). For each fungal strain, different letters indicate significant differences (*p* < 0.05) in their radial growth on the three media (control and supplemented with vanillic or cinnamic acid). * no growth.

Code	Species	Control	Vanillic Acid	Cinnamic Acid
Lt	*Lasiodiplodia theobromae*	3.31 ± 0.07A	3.38 ± 0.15A	3.14 ± 0.08A
Cg	*Colletotrichum gleosporioides*	3.85 ± 0.09A	3.93 ± 0.08A	5.01 ± 0.07B
Fx	*Fusarium oxysporum*	2.70 ± 0.03A	3.15 ± 0.10A	3.32 ± 0.08A
Pp	*Phaeoacremonium parasiticum*	8.80 ± 0.25A	9.52 ± 0.28A	9.88 ± 0.48A
Pi	*Penicillium italicum*	6.22 ± 0.26A	6.79 ± 0.82A	6.63 ± 0.89A
An	*Aspergillus niger*	4.38 ± 0.08A	4.90 ± 0.12A	8.11 ± 1.11B
Ao	*Aspergillus ochraceus*	7.34 ± 0.35A	7.54 ± 0.59A	9.50 ± 1.11B
Aa	*Alternaria alternata*	3.05 ± 0.01A	3.12 ± 0.02A	5.88 ± 0.17B
Tch	*Trichoderma* sp.	3.00 ± 0.01B	1.10 ± 1.00A	3.94 ± 0.10C
Ar	*Athelia rolfsii*	3.00 ± 0.01A	6.31 ± 0.16B	- *
Rx	*Rosellinia necatrix*	4.17 ± 0.09A	4.09 ± 0.08A	7.89 ± 0.27B
Dg	*Diaporthe amygdali*	4.10 ± 0.10A	4.80 ± 0.12A	6.60 ± 0.49B

**Table 3:** Concentrations (as mg/kg) of cinnamic and vanillic acids in controls and media inoculated with 12 different species of fungi. Ctr, control; Ctr-21, control after 21 days.

Ctr	Ctr-21	Cg	Fx	Aa	Tch	Ar	Rx	Lt	Dg	Pp	Pi	An	Ao
Cinnamic acid													
351 ± 7 A	359 ± 10 A, d	363 ± 11 d	364 ± 2 d	363 ± 1 d	363 ± 1 d	355 d	361 ± 1 d	343 ± 7 c	347 ± 1 c	353 ± 2 d	293 ± 23 b	1 a	337 ± 1 c
Vanillic acid													
359 ± 31 A	362 ± 5 A, e	240 ± 4 b	215 ± 30 b	361 ± 1 e	357 ± 5 e	217 ± 9 b	331 ± 13 d	108 ± 16 a	201 ± 59 b	201 ± 11 b	186 ± 25 b	227 ± 51 b	310 ± 8 c

In line, different uppercase letters indicate significant differences between the control and control after 21 days (*p* < 0.05). In line, different lowercase letters indicate significant differences between the control after 21 days and the media inoculated with fungi.
